# CharPlant: A *De Novo* Open Chromatin Region Prediction Tool for Plant Genomes

**DOI:** 10.1016/j.gpb.2020.06.021

**Published:** 2021-03-02

**Authors:** Yin Shen, Ling-Ling Chen, Junxiang Gao

**Affiliations:** 1 Hubei Key Laboratory of Agricultural Bioinformatics, College of Informatics, Huazhong Agricultural University, Wuhan 430070, China; 2 National Key Laboratory of Crop Genetic Improvement, Huazhong Agricultural University, Wuhan 430070, China

**Keywords:** Open chromatin region, Chromatin accessibility, Convolutional neural network, *De novo* prediction, Plant genome

## Abstract

**Chromatin accessibility** is a highly informative structural feature for understanding gene transcription regulation, because it indicates the degree to which nuclear macromolecules such as proteins and RNAs can access chromosomal DNA. Studies have shown that chromatin accessibility is highly dynamic during stress response, stimulus response, and developmental transition. Moreover, physical access to chromosomal DNA in eukaryotes is highly cell-specific. Therefore, current technologies such as DNase-seq, ATAC-seq, and FAIRE-seq reveal only a portion of the **open chromatin regions** (OCRs) present in a given species. Thus, the genome-wide distribution of OCRs remains unknown. In this study, we developed a bioinformatics tool called CharPlant for the ***de novo* prediction** of OCRs in **plant genomes**. To develop this tool, we constructed a three-layer **convolutional neural network** (CNN) and subsequently trained the CNN using DNase-seq and ATAC-seq datasets of four plant species. The model simultaneously learns the sequence motifs and regulatory logics, which are jointly used to determine DNA accessibility. All of these steps are integrated into CharPlant, which can be run using a simple command line. The results of data analysis using CharPlant in this study demonstrate its prediction power and computational efficiency. To our knowledge, CharPlant is the first *de novo* prediction tool that can identify potential OCRs in the whole genome. The source code of CharPlant and supporting files are freely available from https://github.com/Yin-Shen/CharPlant.

## Introduction

In eukaryotic genomes, most of the chromatin regions are tightly coiled in the nucleus, but some regions, known as open chromatin regions (OCRs) or accessible chromatin regions, are loosely formed after chromatin remodeling. Whether the chromatin is loosely or tightly coiled largely determines transcriptional regulation [1], [2]. A number of *cis*-regulatory elements interact with *trans*-acting factors for transcriptional regulation, and *cis*-*trans* elements with regulatory functions participate in the process of transcriptional regulation by binding to OCRs [3], [4]. For example, when a transcription factor binds to an OCR, it recruits other proteins to initiate the transcription of nearby genes. Therefore, a complete genome-wide map of potential OCRs is helpful for the investigation of changes in the nucleosome location and for the discovery of genome regulatory elements and gene regulatory mechanisms [5], [6]. Chromatin accessibility information has even been proven to be valuable for the early diagnosis and treatment of cancer [7], [8].

The OCRs are easier to excise than other regions. Therefore, researchers often use enzymes, such as nuclease and transposase, or physical methods to digest the chromatin. The cleavage-sensitive sites are then sequenced using various technologies, such as DNase I hypersensitive site sequencing (DNase-seq), assay for transposase accessible chromatin sequencing (ATAC-seq), and formaldehyde-assisted isolation of regulatory element sequencing (FAIRE-seq), to obtain further information. DNase-seq has been used for a long time; however, it requires a large amount of starting material (∼ 1 × 10^7^ cells). On the other hand, ATAC-seq requires a significantly smaller sample (< 1 × 10^5^ cells) and has the advantage of requiring no antibody. Therefore, ATAC-seq has become the method of choice in recent years [9], [10]. However, none of these techniques are able to solve the problem of open chromatin determination. All nuclease-based methods exhibit a preference for specific sequences for cleavage, depending on the nuclease, which is a major flaw. For example, DNase I exhibits a strong preference for specific sequences, and many DNase-seq results reflect cleavage preferences rather than actual protein binding [3], [11], [12]. Similarly, in the ATAC-seq method, a preference for cleavage sites has been observed with some of the Tn5 enzymes, resulting in “false DNA footprints” [13]. Although these technologies have been commonly used in human and animal studies [14], their application in plants is still in the exploratory stage. This is because of structural differences between plant and animal cells. Unlike animal cells, plant cells possess a cell wall, numerous chloroplasts, mitochondria, and other organelles that contaminate the assay. Consequently, OCR data have been obtained using DNase-seq and ATAC-seq only in a small number of model plant species, including *Oryza sativa* [15], *Arabidopsis thaliana*, *Medicago truncatula*, *Solanum lycopersicum* [16], and *Hordeum vulgare* [17].

Previous studies have shown that chromatin accessibility is highly dynamic rather than static. OCRs usually change during stress response, stimulus response, and developmental transition [18], [19]. Moreover, OCRs in different species are significantly cell-specific [4]. More than 40% of the OCRs in human T-cells differ between functional and exhausted cells at different time points [20]. Chromatin accessibility also varies considerably among different cells in *Drosophila melanogaster*, *A. thaliana*, and *O. sativa* [16], [21]. Consequently, the current DNase-seq, ATAC-seq, and FAIRE-seq data represent only some of the OCRs and do not present the entire chromatin accessibility information about a given species [22], [23]. Thus, a global overview of the distribution of OCRs in genomes is lacking. Moreover, these experimental technologies are generally expensive and time-consuming [3].

Proteins recognize specific motifs and epigenetic modifications of the DNA sequence that influence its accessibility [24]. After training on a specific dataset, machine-learning algorithms can collect sequence information and predict protein-binding sites, DNA accessibility, histone modifications, and DNA methylation patterns. Many algorithms have been developed to predict regulatory elements, such as Basset [25], Deeperdeepsea [26], DeepBind [27], and DeepCpG [28]. However, these algorithms have a few limitations. First, most of the algorithms are not designed for the prediction of OCRs; instead, they are designed for the prediction of 1) regulatory fragments that bind to transcription factors or RNA-binding proteins, 2) DNase sensitivity, and 3) genomic variants. Second, almost all previous studies using such algorithms are based on human or mouse data; however, the OCRs of plants and animals exhibit significantly different characteristics. For example, approximately 39% of the DNase I hypersensitive sites (DHSs) are associated with introns in the human genome, which is remarkably higher than the proportion of intron-associated DHSs in *O. sativa* (11%) and *A. thaliana* (5%) [29]. Third, most of the existing methods are developed as conventional classifiers that classify sequence fragments of a certain length (hundreds of base-pairs) as regulatory regions, instead of scanning the whole genome.

**Figure 1:**
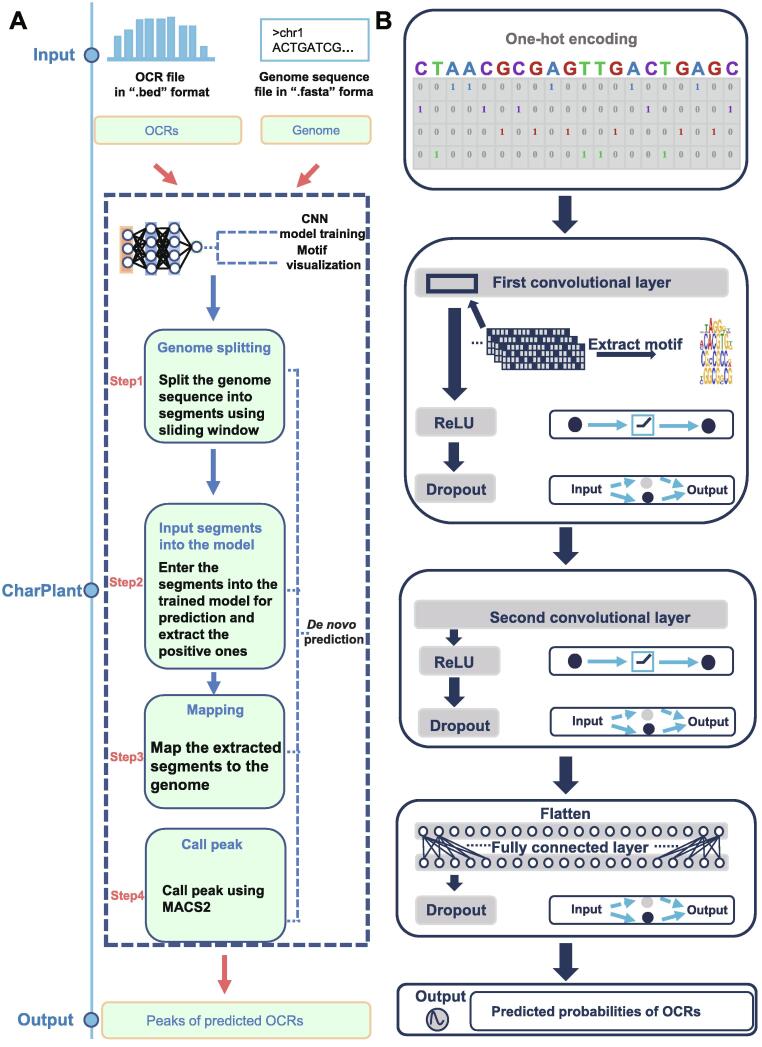
**Steps involved in the construction and execution of CharPlant** **A.***De novo* OCR prediction pipeline. **B.** Construction and training of the CharPlant network. OCR, open chromatin region; CNN, convolutional neural network; ReLU, rectified linear unit.

OCRs are usually rich in various elements and specific motif-binding factors. Therefore, it is feasible to scan the genome and predict chromatin accessibility by learning the motifs and their distribution from OCR data. Here, we developed a *de novo* OCR prediction tool, chromatin accessible regions for plant (CharPlant), based on deep learning to provide a genome-wide overview of OCRs in a given plant species. We constructed training datasets using the DNase-seq and ATAC-seq data of four plant species. CharPlant simultaneously learns the relevant sequence motifs and regulatory logics, which are jointly used to determine DNA accessibility. The trained model accepts DNA sequences or scaffolds as input and generates an outline of OCRs in a “.bed” file as output (Figure 1A). To our knowledge, this is the first tool capable of *de novo* prediction of OCRs from the DNA sequence.

## Method

### Construction of datasets

Considering that a dataset has to be constructed using representative plant species that have both OCR assay data and high-quality reference genomes, the DNase-seq data of *O. sativa* and the ATAC-seq data of four plant species (*A. thaliana*, *S. lycopersicum*, *M. truncatula*, and *O. sativa*) were downloaded from the PlantDHS database at https://plantdhs.org and the Gene Expression Omnibus (GEO) of NCBI (GEO: GSE101482 and GSE75794) at https://www.ncbi.nlm.nih.gov/geo [15], respectively. Detailed information about the DNase-seq and ATAC-seq data is listed in Table 1, and basic information about the reference genomes of these four plant species is listed in Table 2. Because the DNase-seq and ATAC-seq data used here represent diverse plant species of both dicot and monocot lineages and different cell types of the same species (*O. sativa*), this model applies to a broad range of plant species with distant evolutionary relationships. The use of two mainstream technologies, DNase-seq and ATAC-seq, allowed the inclusion of nuclease-based and transposase-based data. MACS2 software was used for peak calling with default parameters [30]. Because of higher statistical power using long fragments, peaks longer than 200 bp were filtered out as positive samples. Positive sequences were shuffled to generate the negative dataset with fasta-shuffle-letters in the MEME software [31]. Unlike the random interception of fragments from the DNA sequence for use as negative samples, shuffling ensures that the negative and positive samples have identical composition of all four bases [27]. Shuffling also maintains a balance between positive and negative samples when constructing the dataset. In an unbalanced dataset, classification algorithms focus on the class containing the most samples, which degrades the classification performance of the class that contains a small number of samples. Most machine-learning algorithms do not work well with unbalanced datasets. Therefore, to construct the dataset in this study, a negative sequence was generated using each positive sequence, *i.e.*, the number of positive and negative samples were equal in number. The samples were divided into three sets, training set, validation set, and testing set, which accounted for 60%, 20%, and 20% of the data, respectively. The sample numbers of the three sets are listed in Table 1.

**Table 1:** **Detailed information on the DNase-seq and ATAC-seq datasets**

**Species**	**Data type**	**Database**	**Accession No.**	**Tissue and treatment**	**Sample number**		
					**Training set**	**Validation set**	**Testing set**
*Oryza sativa*	DNase-seq	PlantDHS	NA	Seedlings and calli	70,810	23,604	23,604
*Oryza sativa*	ATAC-seq	NCBI	GSE101482	Roots of 7-day-old seedlings; two biological replicates	−	−	39,868
*Oryza sativa*	ATAC-seq	NCBI	GSE75794	2nd leaves of 14-day-old seedlings; heat stress and recovery, dehydration stress and recovery	−	−	30,634
*Arabidopsis thaliana*	ATAC-seq	NCBI	GSE101482	Root cells; two biological replicates	13,778	4594	4596
*Solanum lycopersicum*	ATAC-seq	NCBI	GSE101482	Root cells; two biological replicates	32,168	10,724	10,726
*Medicago truncatula*	ATAC-seq	NCBI	GSE101482	Root cells; two biological replicates	27,982	9328	9330

*Note*: “−” represents that all GSE101482/GSE75794 data were used as the testing set.

**Table 2:** **Genome-related information on the four plant species used in this study**

**Species**	**Genome size (bp)**	**Database**	**Website**
*Oryza sativa*	373,245,519	IRGSP-1.0	RAP–DB (https://rapdb.dna.affrc.go.jp)
*Arabidopsis thaliana*	119,667,750	TAIR10	TAIR (https://www.arabidopsis.org)
*Solanum lycopersicum*	823,944,041	ITAG2.4	Phytozome (https://phytozome.jgi.doe.gov)
*Medicago truncatula*	384,466,993	MT4.0	*Medicago truncatula* genome database (https://www.medicagogenome.org)

*Note*: RAP–DB, Rice Annotation Project Database; TAIR, The *Arabidopsis* Information Resource.

### Construction of the CharPlant model

The CharPlant model is trained using ATAC-seq data of four plants or DNase-seq data of *O. sativa*, and each plant has its own model parameters. The model is based on a multilayer convolutional neural network (CNN) (Figure 1B). The CNN model, which originates from artificial neural networks, contains perceptrons with multiple hidden layers and combines low-level features to form more abstract high-level attributes or features for discovering feature representations of data [32], [33]. The motivation is to build a neural network that can simulate the human neuron for analysis and learning, and imitate the mechanism of the human brain to interpret data, such as images, sounds, and texts [34], [35], [36]. Unlike traditional methods, in which features are manually selected in the pre-processing stage, the CNN adaptively extracts features from large-scale training datasets. It then maps input data to high-dimensional representations with abundant information by nonlinear transformation, thus simplifying classification or regression. Early application of the CNN model in DNA sequence analysis surpasses existing mature algorithms, such as support vector machines or Random forests, in predicting protein binding and DNA sequence accessibility [25], [27].

To achieve high computational efficiency, our model is designed with only three hidden layers: the first and second layers are convolutional, whereas the third layer is fully connected. The CNN model used in CharPlant requires binary vectors as input. Each input DNA fragment is first converted into a 4 × *n* matrix, where *n* represents the length of the input fragment. Thus, each base is preprocessed with “one-hot” encoding (A: [1, 0, 0, 0]; C: [0, 1, 0, 0]; G: [0, 0, 1, 0]; T: [0, 0, 0, 1]; N: [0, 0, 0, 0]), and the sequence is converted into a matrix with four rows. The first layer of the CNN model contains convolutional filters for the identification of low-level features in a given DNA sequence. A convolutional filter is an essential motif prober that scans each input matrix to discover potential patterns. The identification of low-level DNA features involves the following steps. First, each input sequence is fed into the first convolutional layer, and the convolution kernel slides over the sequence fragment to calculate the activation score. If the activation score of the convolution kernel at a certain position is greater than the preset threshold, the sequence segment centered at that position will be identified and represented by the position frequency matrix (PFM) of four base frequencies. Then, the PFM is used to calculate the information entropy and is transformed into position weight matrix (PWM), which is widely used for the representation of motifs. The PMW contains four rows, and describes the entropy of four bases at each position [25], [37]. Subsequently, the sequence logo is used to visualize the motif, *i.e.*, the base size of each position indicates the possibility of the base at this position. To obtain the activation score of the convolutional filter, the rectified linear unit (ReLU) is used as the activation function for three hidden layers. The ReLU function *f*(*x*) is calculated as follows:

$$f\left(x\right)=max\left(0,x\right)$$

where *x* is the input.

The ReLU function is used by neurons just like traditional activation functions such as sigmoid or hyperbolic tangent. Compared with the conventional activation function, ReLU has much less computational complexity for calculating the error gradient in back propagation. Additionally, when the conventional activation function propagates backward, it is likely that the derivative will approach zero, which would make it impossible to complete the training of deep network. However, ReLU overcomes this shortcoming very well.

To overcome the problem of over-fitting, random dropout is set after every hidden layer in the model. Dropout is an optimization method for resolving over-fitting and gradient disappearance in deep neural networks. In the learning process of the neural network, the weight of randomly selected nodes in the hidden layer is set at zero. Because different nodes are reset to zero after each iteration, the importance of each node is balanced. Because of the use of random dropout, each node of the neural network contributes roughly equally to the training, and there is no case where a few high-weight nodes completely dominate the output. In this study, the dropout probability is set at 0.6, *i.e.*, the weights of 60% of the neurons are set to zero in every iteration.

The architecture of the second layer is the same as that of the first layer and is based on three key technologies: convolutional network, ReLU activation function, and dropout. The second layer combines low-level motif features to form abstract high-level attributes. In the CNN model, a fully connected layer is set behind the two convolutional layers. Each neuron in the fully connected layer is connected with all the neurons in its preceding layer to integrate local sequence information with class discrimination in the convolutional layer. The fully connected layer contains 200 neurons, and it flattens the matrix into a column vector. The weights of links are calculated by a linear regression algorithm, but linear regression could only predict the continuous value, which does not solve the classification problem. Therefore, the output layer determines whether the input sequence belongs to the positive or negative class, depending on the calculations. The final output layer uses sigmoid function to perform nonlinear transformation and maps the results of the fully connected layer from (−∞, +∞) to (0, 1), which indicates the probability of open chromatin sequence. The goal of the training model is to minimize the error between predicted and labeled values, *i.e.*, to minimize the cost function. A series of cost functions is available. Here, the binary cross-entropy cost function is used because it can overcome the problem of gradient disappearance when calculating gradient descent, thus showing high learning efficiency.

### Implementation of CharPlant

CharPlant, implemented in Python, is based on Keras 2.0.1 with TensorFlow 1.2.0, an open source machine-learning platform developed by Google. Additionally, a widely used workflow management system, Snakemake, is employed to combine a series of steps into a single pipeline that can be run by an inexperienced user using simple command line entries [38]. Steps in the workflow are described in terms of the rules defined using the input and output and Shell and Python codes. The workflow determines the steps that need to be performed and produces one or more output files. Dependencies between rules are automatically resolved, and rules are automatically parallelized when possible. A text file titled “Snakefile” is created, which defines the input and how the output is created from the input. CharPlant can learn OCR features from DNase-seq or ATAC-seq data and predict potential chromatin accessible regions in a plant genome *de novo*. It performs four steps: 1) data pre-processing, 2) model training, 3) motif visualization, and 4) *de novo* prediction. If all the steps are successful, CharPlant outputs the results of the predicted OCRs in a “.bed file” in the directory CharPlant/peak.

Snakefile has a number of parameters, such as epoch number, learning rate, batch size, and dropout, which could be adjusted using a configuration file “config.yaml”. This configuration file provides default values and their meaning for each parameter. It is not necessary for the users to modify the default values, except for two directories as follows: “genome: Yourpath/CharPlant/example/oryza_sativa.fa” and “bed: Yourpath/CharPlant/example/oryza_sativa.bed”. For example, the parameter “genome” represents the input genome file in “.fasta” format, and the parameter “bed” represents the output OCR file in “.bed” format. The user would need to replace “Yourpath” with the true path in which CharPlant is installed.

### Installation and execution of CharPlant

CharPlant is currently available for Linux-based operating systems. To install and run CharPlant, download the package from the GitHub development platform at https://github.com/Yin-Shen/CharPlant and then set “CharPlant” as the current directory. The subdirectory CharPlant/example contains the reference genome (file “oryza_sativa.fa”) and the DNase-seq data of *O. sativa* as an example (file “ory_whole.bed”). All Python and Shell scripts are in the subdirectory CharPlant/src. Some fundamental Python packages, such as numpy, matplotlib, and keras, will be needed for scientific computing and network construction. File S1 provides a detailed CharPlant manual, installation steps, and parameter settings for the abovementioned Python packages, and complete “config.yaml” and “Snakemake” files. Users can run the program by typing the following command: $ CharPlant.sh.

## Results and discussion

### Motifs identified by CharPlant

**Figure 2:**
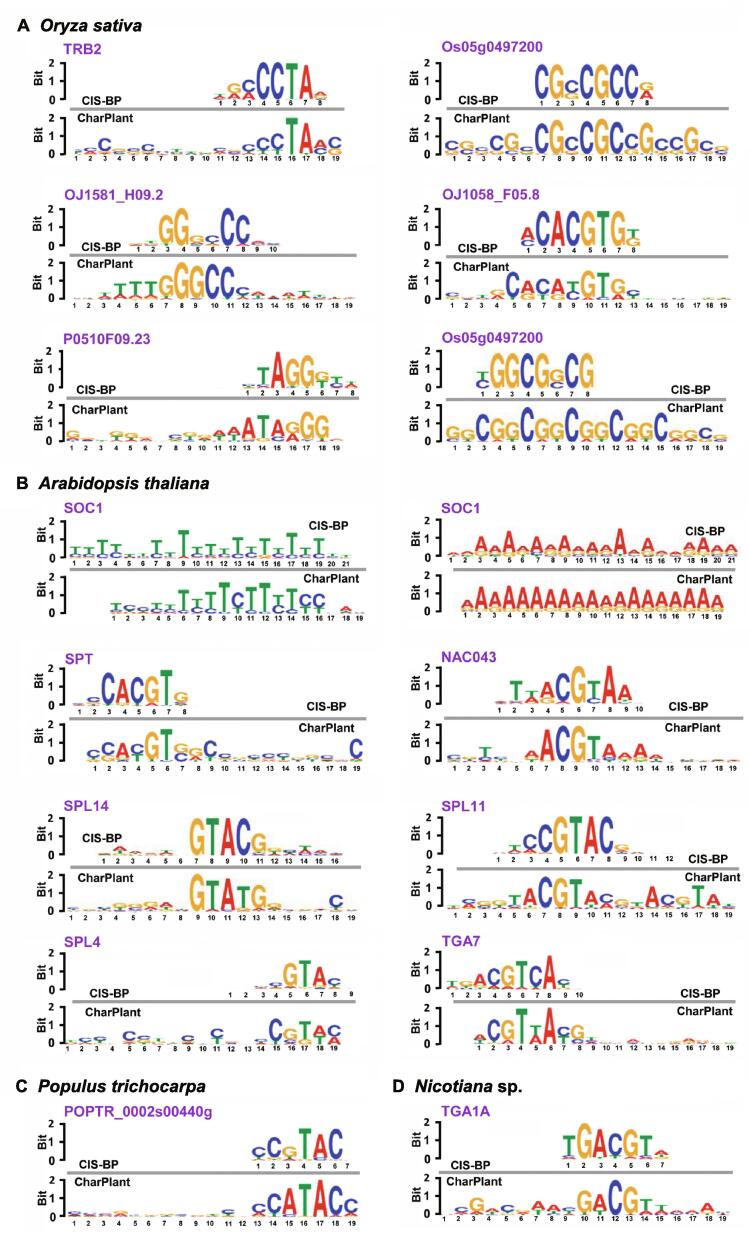
**Motifs identified by CharPlant in *Oryza sativa* and other plant species** **A.** Six of the eight known *Oryza sativa* motifs in the JASPAR database identified by CharPlant. **B.**–**D.** Motifs of *Arabidopsis thaliana* (B), *Populus trichocarpa* (C), and *Nicotiana* sp. (D) in JASPAR identified by CharPlant. CIS-BP, Catalog of Inferred Sequence Binding Preferences.

The positive dataset was obtained from the peaks of ATAC-seq and DNase-seq data, and the negative samples were generated by shuffling the positive samples, as described above. In the positive samples, motifs are usually clustered for protein binding, whereas the negative samples generally have far fewer motifs. A DNA motif is defined as a short similar recurring pattern of nucleotides, with many biological functions. A previous study has shown that sequence motifs are roughly constant in length, and are often repeated and conserved [39]. Based on the difference between positive and negative datasets, our model learned the sequence motifs and the regulatory logics with which they are combined to determine DNA accessibility. The convolutional layer searched for the motifs along the genome sequence and produced a matrix, with rows representing neurons and columns representing positions. Determination of OCRs was based on the accurate identification of motifs. We compared the motifs learned by the convolution kernels with the known motifs in the JASPAR database [37]. The results showed that many of the motifs predicted by our model were previously known and experimentally validated. For example, JASPAR has eight known motifs in *O. sativa*, of which six were identified by our model (Figure 2A). We also identified many known motifs of other plants, some of which are shown in Figure 2B–D. Notably, sequence motifs were very small in size (6–19 bp), whereas intergenic regions were very long and highly variable, thus making motif discovery a very difficult task. Therefore, the number of motifs in plant genomes and their positions with respect to target genes remain unclear. Given that JASPAR has a very limited repertoire of only 501 experimentally validated motifs, some of the identified sequences not included in the database could be potential motifs, which might be experimentally validated in the future. Overall, CharPlant can detect motifs of various lengths, which can be subsequently used for the identification of OCRs.

### Performance comparison between CharPlant and other methods

**Figure 3:**
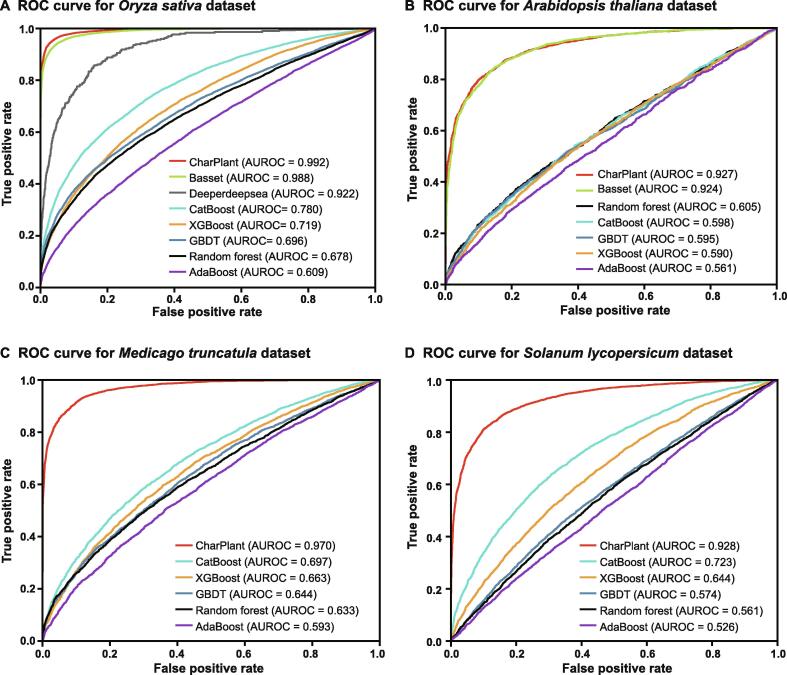
**Comparison of ROC curves and AUROC values between CharPlant and comparative methods on four plant datasets** **A.***Oryza sativa*. **B.***Arabidopsis thaliana*. **C.***Medicago truncatula*. **D.***Solanum lycopersicum*. ROC, receiver operating characteristic; AUROC, area under the ROC curve.

CharPlant is designed as a *de novo* OCR prediction tool. By contrast, almost all current methods are developed as conventional classifier algorithms and cannot scan the genome sequence to discover OCRs. Moreover, the architecture and parameters of these models are developed based on human and animal data. Therefore, a strict comparison of these methods with CharPlant is difficult. Because it was impossible to compare *de novo* prediction with other methods, we compared the learning ability and computational efficiency of CharPlant with two state-of-the-art deep learning algorithms, Basset [25] and Deeperdeepsea [26], using chromatin accessibility data of plants. Basset is an open source package and learns the functional activity of DNA sequences from genomic data. The authors applied Basset to a compendium of accessible genomic sites mapped in 164 cell types by DNase-seq and showed greater predictive accuracy than previous methods [25]. We revised Basset to adapt it to plant data. Deeperdeepsea is a recently published PyTorch-based deep learning library for any biological sequence data. We downloaded the package from https://selene.flatironinstitute.org/. Each method was adjusted to its best state and trained using the dataset constructed in this study, as described above. We calculated the false positive rate *vs.* true positive rate to plot receiver operating characteristic (ROC) curves and determined the area under the ROC curve (AUROC; Figure 3, Figure S1). In Figure 3A, the curves were obtained using the DNase-seq data of *O. sativa*. Although the AUROC values of CharPlant were slightly better than those of Basset and Deeperdeepsea on the *O. sativa* DNase-seq data, no major difference was observed among the three methods. However, the performances of these three methods on the datasets of other three species (*A. thaliana*, *M. truncatula*, and *S. lycopersicum*) were quite different (Figure 3B–D). Although the ROC curves and AUROC values of Basset on *O. sativa* and *A. thaliana* datasets were similar to those of CharPlant, the prediction accuracy of Basset was only ∼ 50% with *S. lycopersicum* and *M. truncatula* datasets. Thus, the results of Basset were equivalent to a random guess, indicating that Basset failed to predict OCRs. Similarly, Deeperdeepsea failed on the datasets of all analyzed plant species, except *O. sativa.* By contrast, our method could be applied to all datasets and achieve consistent performance. Basset and Deeperdeepsea are both excellent methods for the prediction of regulatory elements and have been proven to produce accurate results after training on human data. However, these methods do not work on plant datasets, as shown in this study. This is likely because the structural design and hyperparameter choice of the model are not suitable for plant datasets. The characteristics of DNA sequences differ greatly between plants and animals. To shift an algorithm from the animal to the plant system, replacing the animal training set with a plant dataset is not sufficient; instead, to achieve similar performance, it is often necessary to make substantial changes to the model structure, essentially transforming it into a new model.

To further validate the performance of CharPlant, we compared our model with machine-learning methods including Random forest, Adaboost, GBDT, XGBboost, and CatBoost on all four plant datasets. These machine-learning methods were implemented using the Scikit-learn package, a widely used library that supports supervised and unsupervised learning [40]. We computed the precision and recall ratios of the comparative methods, and plotted the precision recall (PR) curves. As shown in Figure S2, analysis of the PR curves of CharPlant, Basset, and Deeperdeepsea led us to a similar conclusion to that obtained from the analysis of ROCs described above (Figure 3). When the other five comparative methods, including Random forest, Adaboost, GBDT, XGBboost, and CatBoost, were used on the four plant datasets, their performances were similar, and their ROC and PR curves were close to each other. However, the performance of each of these algorithms was significantly inferior to that of neural network methods.

In some instances, the method of sample partitioning influences model evaluation. To avoid the randomness of a single training set and testing set, we performed 10-fold cross validation. The dataset was divided into 10 parts and each part was used in turn as a testing dataset, and nine were used as the training dataset. ROC and PR curves were plotted for each test (Figure S3). All samples were used as training and testing sets, and each sample was tested one time. The majority of PR curves overlapped, indicating that precision and recall were stable when using different dataset partition methods. Similar conclusions could be drawn from ROC curves.

To compare the computation efficiencies, we trained and tested CharPlant, Basset, and Deeperdeepsea on the central processing unit (CPU) and graphics processing unit (GPU). The manufacturers and models are as follows: Tesla P100-PCIE-16GB (GPU) and Intel(R) Xeon(R) Gold 6140 CPU @ 2.30 GHz (CPU). The comparison was performed on the DNase-seq dataset of *O. sativa*. The results showed that CharPlant took significantly less time than Basset and Deeperdeepsea on both CPU and GPU (Table 3).

**Table 3:** **Computation efficiency of the three methods on CPU and GPU**

**Method**	**Training time (s)**		**Testing time (s)**	
	**GPU**	**CPU**	**GPU**	**CPU**
CharPlant	2558	280,640	21	2371
Basset	3205	382,093	28	3533
Deeperdeepsea	5520	13,502,400	290	12,465

*Note*: CPU, central processing unit; GPU, graphics processing unit.

### ***De novo*** prediction of OCRs in genomes

**Figure 4:**
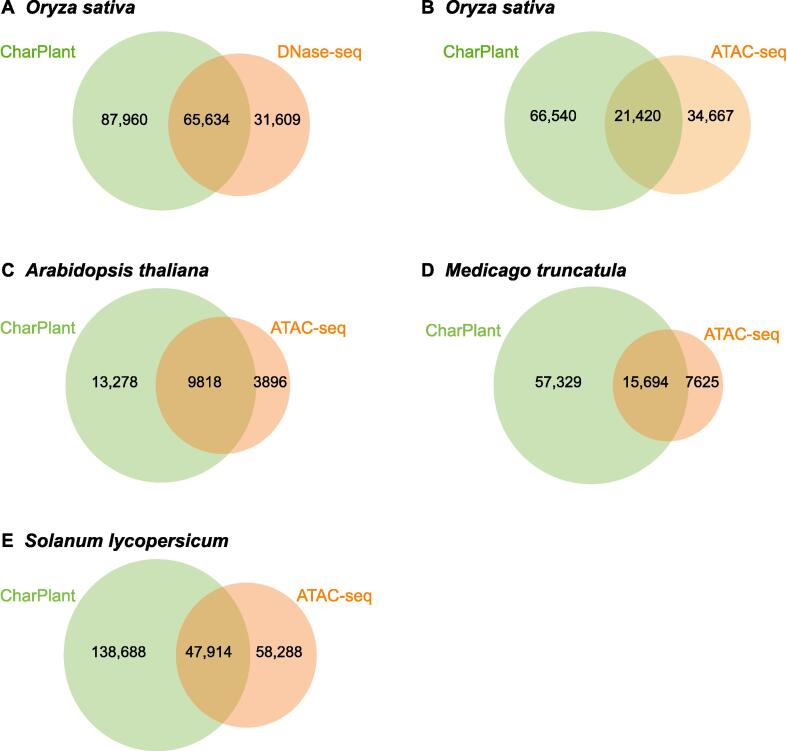
**Overlap between OCRs predicted by CharPlant and those detected by DNase-seq or ATAC-seq assays in four plant species** **A.** Overlap between the CharPlant-predicted OCRs and those detected by DNase-seq assay in *Oryza sativa*. **B.** CharPlant-predicted OCRs supported by ATAC-seq assay after excluding the overlap with DNase-seq assay. ATAC-seq data were from GSE101482 and GSE75794. **C.**–**E.** Overlap between the CharPlant-predicted OCRs and those detected by ATAC-seq assay in *Arabidopsis thaliana* (C), *Medicago truncatula* (D), and *Solanum lycopersicum* (E).

To enable the prediction of OCRs from long DNA sequences or complete genomes using CharPlant, we used the sliding-window method to split the sequence into fragments. The window width was set at 36 bp. Generally, a smaller sliding step is helpful for the accurate prediction of the locations of OCRs; however, the computational complexity with a smaller sliding step is significantly higher than that with a large sliding step. To compromise the calculation efficiency and accuracy, the sliding step was set at 5 bp. The trained model was used to calculate the probability of chromatin accessibility of these fragments, and then the peaks of OCRs in these fragments were called using the MACS2 tool, with default parameters [30]. We scanned the whole genome sequences of four plant species using the CharPlant model and aligned the predicted OCRs with the DNase-seq or ATAC-seq dataset to validate the performance of CharPlant. The training datasets were obtained from a single cell type at a specific time, yet we tried to predict all potential OCRs in different tissues at different times. The results showed that the number of OCRs in the latter was higher than that in the former. Notably, the number of OCRs predicted by CharPlant in all datasets was much larger than that detected by DNase-seq or ATAC-seq assays (Figure 4). CharPlant predicted 153,594 potential OCRs in the DNase-seq dataset of *O. sativa* seedlings and calli, of which 65,634 overlapped with those detected by the DNase-seq assay (Figure 4A). Although the remaining 87,960 predicted OCRs were not supported by the DNase-seq assay, 21,420 of these were supported by the ATAC-seq assay of *O. sativa* roots and leaves (ATAC-seq data from GSE101482 and GSE75794) (Figure 4B). Based on the currently available DNase-seq and ATAC-seq data of a few plant species, it is reasonable to speculate that more predicted OCRs could be confirmed if more experimental data were available. Among the OCRs predicted in the *O. sativa* seedling/callus data, a considerable proportion was supported by the root/leaf data, implying that the OCRs predicted by CharPlant are credible and not false positives.

**Figure 5:**
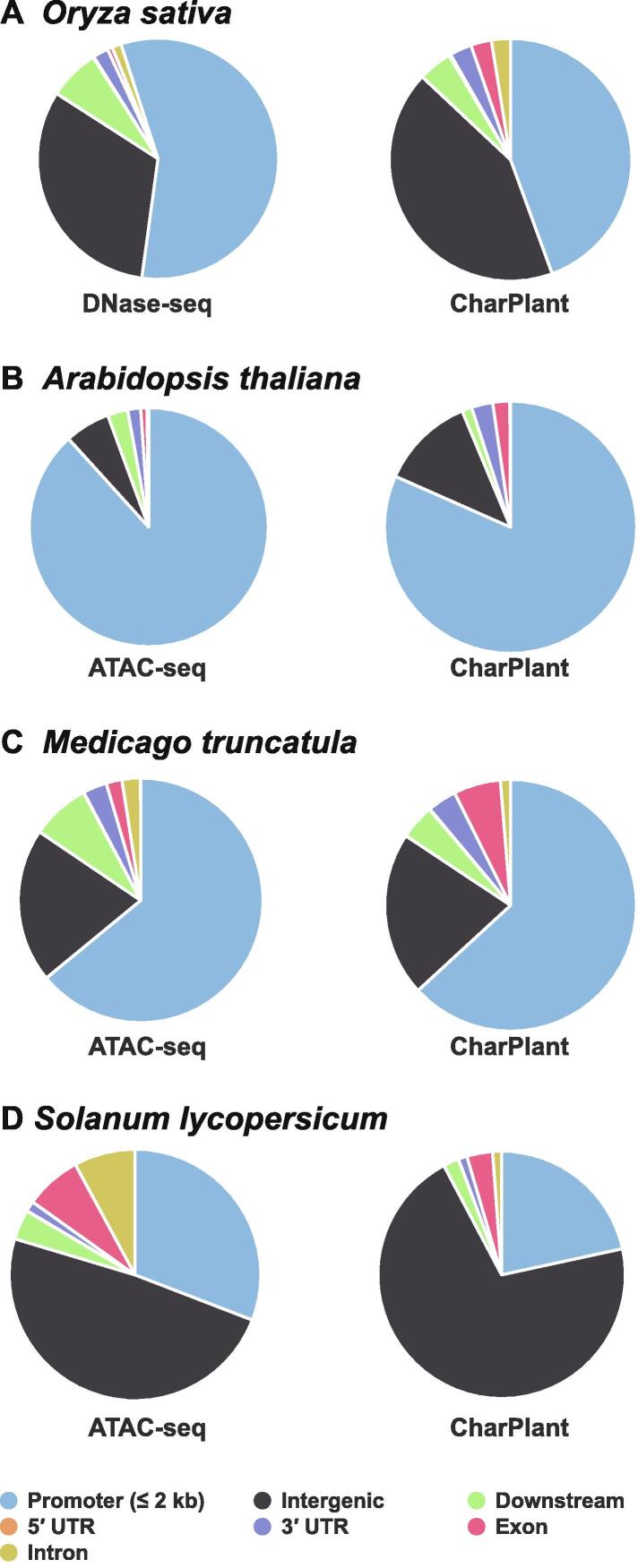
**Distributions of CharPlant-predicted OCRs and experimental OCRs in four plant species**

To provide more evidence, we compared the predicted OCRs with experimental OCRs and three types of histone modifications (H3K4me3, H3K9ac, and H3K27ac) in *A. thaliana*. Covalent modification of the histone tail plays a key role in regulating chromatin structure and gene transcription. In eukaryotes, H3K4me3 is associated with active chromatin and promotes transcription through interactions with effector proteins [41], [42]. H3K9ac and H3K4me3 frequently coexist as markers of active gene promoters. H3K27ac is related to gene activation and is mainly enriched in enhancer and promoter regions [43], [44]. Figure S4A and B show two examples of overlap between predicted OCRs and experimental OCRs, indicating that the ATAC-seq data supported the predicted results. Additionally, H3K4me3, H3K9ac, and H3K27ac modifications showed peaks at these sites. However, another scenario is that a predicted OCR does not overlap with DNase-seq or ATAC-seq data. For example, as shown in Figure S4C, the ATAC-seq data showed no peak at the predicted OCR, whereas H3K4me3, H3K9ac, and H3K27ac modifications showed significant peaks. Considering the tissue- and time-specificity of OCRs, it is difficult to definitively conclude that this site is not an OCR. To determine whether there is a prediction bias, *i.e.*, some regions have higher prediction accuracy than other regions, we calculated the distributions of predicted OCRs in the promoter (≤ 2 kb) regions, intergenic regions, exons, introns, 5′ UTRs, 3′ UTRs, and downstream regions (300 bp downstream of transcription termination sites), and compared them with experimental data to show their consistency. The results showed that the distributions of predicted OCRs were consistent with those of the DNase-seq data in *O. sativa* and the ATAC-seq data in *A. thaliana* and *M. truncatula* (Figure 5A–C). Although the distribution of predicted OCRs appeared different from that of the ATAC-seq data in *S. lycopersicum*, the number of OCRs was highest in the intergenic regions, followed by the promoter regions, and least in the 3′ UTRs (Figure 5D). Epigenetic modifications provide further evidence for the validation of OCRs predicted by CharPlant. Among the four plant species, *A. thaliana* has the most abundant data, including ATAC-seq dataset and various epigenetic datasets. Therefore, we used *A. thaliana* as an example to compare the difference in the frequency of H3K4me3 modification between the predicted OCRs and ATAC-seq peaks. The distribution of H3K4me3 in the *A. thaliana* genome was obtained from the Plant Chromatin State Database (PCSD; https://systemsbiology.cau.edu.cn/chromstates) [45]. Our results showed no significant difference in the frequency of H3K4me3 modification between the predicted OCRs and ATAC-seq peaks, and the two boxplots were almost identical (Figure S5A). Furthermore, we investigated the difference of H3K4me3 modification between the predicted OCRs and 10,000 randomly selected inactive chromatin regions (based on ATAC-seq data). We found that the predicted OCRs were significantly more enriched for H3K4me3 modification than the unopened chromatin regions (Figure S5B).

Notably, the time taken to scan the genome of four plant species was closely related to the genome size; analyses of *A. thaliana*, *O. sativa*, *M. truncatula*, and *S. lycopersicum* genomes took 8 h, 22 h, 24 h, and 49 h, respectively.

## Conclusion

In summary, experimental technologies can determine only the current status of DNA accessibility, whereas CharPlant is a neural network model that learns the sequence motifs and regulatory logics and predicts potential OCRs, according to the experimental data. Compared with existing algorithms, CharPlant has several advantages. First, to our knowledge, CharPlant is the first *de novo* prediction tool that can identify potential chromatin accessible regions along the genome sequence. Second, CharPlant is specifically designed to predict OCRs of plants, rather than those of human or animals, as in other algorithms. Third, CharPlant marks all potential OCRs of a given plant species in different tissues and at different times, which is beneficial for the investigation of gene regulation under different conditions. Lastly, CharPlant is significantly faster than other deep learning algorithms because it is designed with a concise and efficient structure.

## Code availability

The source code of CharPlant and supporting files are freely available from GitHub at https://github.com/Yin-Shen/CharPlant.

## CRediT author statement

**Yin Shen:** Methodology, Data curation, Software. **Ling-Ling Chen:** Conceptualization, Supervision. **Junxiang Gao:** Conceptualization, Project administration, Validation, Writing - original draft, Writing - review & editing. All authors have read and approved the final manuscript.

## Competing interests

The authors have declared no competing interests.

## Supplementary material

Supplementary File S1 **Steps involved in the installation and execution of CharPlant, config.yaml file, and Snakemake file**.

Supplementary Figure S1 **ROCs of Oryza sativa ATAC-seq data** ROC, receiver operating characteristic; AUC, area under the ROC curve.

Supplementary Figure S2 **Precision recall curves of comparative methods in four plant species A.***Oryza sativa*. **B.***Arabidopsis thaliana*. **C.***Medicago truncatula*. **D.***Solanum lycopersicum*. PR, precision recall.

Supplementary Figure S3 **Receiver operating curves and precision recall curves of 10-fold cross validation A.** Receiver operating curves. **B.** Precision recall curves. ROCs, receiver operating curves; PR, precision recall.

Supplementary Figure S4 **Comparison of predicted OCRs, experimental OCRs, and histone modifications H3K4me3, H3K9ac, and H3K27ac in *Arabidopsis thaliana* A.** and **B.** Two examples showing overlap between predicted OCRs and experimental OCRs. **C.** An example where ATAC-seq data showed no peak at the predicted OCR, but H3K4me3, H3K9ac, and H3K27ac modifications showed significant peaks. OCR, open chromatin region.

Supplementary Figure S5 **Difference in the epigenetic modification H3K4me3 in *Arabidopsis thaliana* A.** Between the open chromatin regions predicted by CharPlant and ATAC-seq peaks. **B.** Between the open chromatin regions predicted by CharPlant and randomly selected inactive regions.
