# Supplementary material for: CharPlant: A De Novo Open Chromatin Region Prediction Tool for Plant Genomes
Source: Genomics Proteomics Bioinformatics. 2021 Mar 2;19(5):860–71. doi: 10.1016/j.gpb.2020.06.021 (PMC9170768; doi:10.1016/j.gpb.2020.06.021)
Supplement: Supplementary File S1 — Steps involved in the installation and execution of CharPlant, config.yaml file, and Snakemake file. [file mmc1.docx]

**File S1**

**Steps involved in the installation and execution of CharPlant, config.yaml file, and Snakemake file**

Note N1: Steps to install and run CharPlant.

Note N2: Complete config.yaml file for helping users understand CharPlant in depth.

Note N3: Complete Snakemake file for helping users understand CharPlant in depth.

**Note** N**1: Steps to install and run CharPlant.**

Follow steps 1 and 2 to install needed packages, and set up a running environment for the software. Both steps are optional for users who have previously installed and executed these packages. Follow Step 3 to download and run CharPlant. Detailed instructions are provided to run CharPlant, although it can be run using a simple command line. In the following description, prompt “$” starts a shell command line of Linux, while the symbol “#” starts a comment.

**Step 1. Install Python packages** (optional for users who have previously installed and executed these packages).

CharPlant was developed using Python. Therefore, some fundamental packages are indispensable for scientific computing and network construction. To efficiently install and manage these packages, we strongly recommend installing the “Conda” package manager first.

(i) Install Conda

$ wget https://repo.continuum.io/archive/Anaconda3-4.2.0-Linux-x86_64.sh
$ bash Anaconda3-4.2.0-Linux-x86_64.sh
$ echo "export PATH=\"${PWD}/anaconda3/bin:\$PATH\" " >> ~/.bashrc
$ source ~/.bashrc

(ii) Install needed Python packages.

The following Python packages are required:

numpy

matplotlib

pyfiglet

sklearn

keras

tensorflow-gpu

(iii) Create environments.

$ conda create --name charplant-cpu python=3.6

$ source activate charplant-cpu

$ pip install sklearn

$ pip install matplotlib

$ pip install pyfiglet

$ conda install keras

$ source deactivate charplant-cpu

**Step 2. Install Bowtie2 , MACS2, and Snakemake** (optional for users who have previously installed and executed these packages).

(i) Bowtie2 (version 2.2.6; for sequence alignment) can be downloaded from <https://sourceforge.net/projects/bowtie-bio/files/bowtie2/>.

$ wget https://downloads.sourceforge.net/project/bowtie-bio/bowtie2/2.2.6/bowtie2-2.2.6-linux-x86_64.zip
$ unzip bowtie2-2.2.6-linux-x86_64.zip
$ echo "export PATH=\"${PWD}/bowtie2-2.2.6:\$PATH\" " >> ~/.bashrc
$ source ~/.bashrc

(ii) MACS2 (version 2.1.1; for peak calling) can be downloaded from <https://pypi.org/project/MACS2/2.0.10.20130915/#history>.

$ wget https://files.pythonhosted.org/packages/0f/e9/60761f3df0634a50a96531fdb00f45dd3a6f3aa2509fb722eb2a665faf35/MACS2-2.1.1.20160226.tar.gz
$ tar -zxvf MACS2-2.1.1.20160226.tar.gz
$ conda create --name macs2 python=2.7
$ source activate macs2
$ cd MACS2-2.1.1.20160226 
$ pip install numpy 
$ python setup.py install 
$ source deactivate macs2

(iii) Snakemake, which combines a series of steps into a single pipeline, can be obtained from <https://snakemake.readthedocs.io/en/stable/index.html>.

$ pip install snakemake

**Step 3. Download and run CharPlant.**

(i) Download and install CharPlant.

Download CharPlant from <https://github.com/Yin-Shen/CharPlant> and set “CharPlant” as current directory. All Python and shell scripts can be found in the subdirectory “CharPlant/src”.

$ git clone https://github.com/Yin-Shen/CharPlant.git
$ cd CharPlant
$ echo "export PATH=\"${PWD}/CharPlant:\$PATH\" " >> ~/.bashrc
$ source ~/.bashrc

To obtain help, use the following command line:

$ CharPlant.sh –h

(ii) Set the parameters of CharPlant.

Configuration file “config.yaml” contains all parameters of CharPlant. To run CharPlant, users need to revise three parameters according to their path and file name as follows:

#Genome file in .fasta format for input (Need full path)
genome: Yourpath/CharPlant/example/oryza_sativa.fa

#Open chromatin regions file in .bed format for input (Need full path)
bed: Yourpath/CharPlant/example/oryza_sativa.bed

#Prefix of the output file
out: ory

(iii) Run CharPlant.

The Snakemake file defines the rules to perform various operations. It is not necessary for the users to rewrite this file.

$ CharPlant.sh

(iv) Output files.
The results will be output into the following five directories:

/data_preprocessing—Contains data preprocessing results for model training and motif visualization.

/model—Contains .json file of model architecture, .h5 file of model parameters, and .png file of the result.

/motif—Contains positional weight matrix of motifs.
/de_novo_prediction—Contains results of *de novo*prediction.
/peak—Contains peaks of predicted OCRs.

**Note N2: Complete config.yaml file for helping users understand CharPlant in depth.**

#Data preprocessing

#Genome file in fasta format(Need full path)

genome: Yourpath/CharPlant/example/oryza_sativa.fa

#Bed file of Chromatin Accessible Regions for input(Need full path)

bed: Yourpath/CharPlant/example/oryza_sativa.bed

out: ory #Prefix of the output file

#model_parameter (Default or custom parameters may be used)

epochs: 150 #Number of epochs (default = 150)

patience: 20 #Number of epochs for early stopping (default = 20)

learningrate: 0.001 #Learning rate (default = 0.001)

batch_size: 128 #Batch size (default = 128)

dropout: 0.6 #Dropout rate (default = 0.6)

#Number of filters in the first convolutional layer (default = 200)

nb_filter1: 200

#Number of filters in the second convolutional layer (default = 100)

nb_filter2: 100

#Length of filters in the first convolutional layer (default = 19)

filter_len1: 19

#Length of filters in the second convolutional layer (default = 11)

filter_len2: 11

#Units in the third fully connected layer (default = 200)

hidden: 200

#motif

motif_out: Ory #Prefix of motif output folder (example:Ory_motif)

#de_novo_prediction

prediction_out: split_fasta_36_1 #Prefix of the output file

#Number of lines split genome file into smaller files (default = 20,000)

split_lines: 20000

#Threshold adopted to assign positive predictions (default = 0.5)

threshold: 0.5

#batch_submit_job

#How the Python script runs in batches;

#examples: local; LSF (LSF Cluster); PBS (PBS Cluster)

run_type: local

#Number of tasks for a job array in one epoch;

batch_submit_jobs_number: 40

#get_positive_sample

#For generating the final fasta file.

speices_name: Rice

#bowtie2_index

index_name: rice #Index name used in bowtie2

#bowtie2

#Sam file prefix (example: whole_fasta_36_1)

sam_prefix: whole_fasta_36_1

#macs2

peak_prefix: *Oryza_sativa* #Call peak file prefix

**Note N3: Complete Snakemake file for helping users understand CharPlant in depth.**

configfile: "config.yaml"

rule all:

rule data_preprocessing:

input:

genome=expand("{sample}", sample=config["genome"]),

bed=expand("{sample}", sample=config["bed"]),

params:

out=config["out"]

priority: 100

shell:

"mkdir $PWD/data_preprocessing && cd $PWD/data_preprocessing && python ../src/data_preprocess.py -g {input.genome} -b {input.bed} -o {params.out} 2> /dev/null"

rule model_training:

params:

epochs=config["epochs"],

patience=config["patience"],

learningrate=config["learningrate"],

batch_size=config["batch_size"],

dropout=config["dropout"],

nb_filter1=config["nb_filter1"],

nb_filter2=config["nb_filter2"],

filter_len1=config["filter_len1"],

filter_len2=config["filter_len2"],

hidden=config["hidden"]

priority: 90

shell:

"mkdir $PWD/model && cd $PWD/model && python ../src/model.py -e {params.epochs} -p {params.patience} -lr {params.learningrate} -b {params.batch_size} -d {params.dropout} -n1 {params.nb_filter1} -n2 {params.nb_filter2} -fl1 {params.filter_len1} -fl2 {params.filter_len2} -hd {params.hidden} 2> /dev/null"

rule motif:

params:

nb_filter1=config["nb_filter1"],

filter_len1=config["filter_len1"],

motif_out=config["motif_out"]

priority: 80

shell:

"mkdir $PWD/motif && cd $PWD/motif && python ../src/motif.py -n1 {params.nb_filter1} -fl1 {params.filter_len1} -o {params.motif_out} 2> /dev/null"

rule de_novo_prediction:

input:

genome=expand("{sample}", sample=config["genome"]),

params:

prediction_out=config["prediction_out"],

split_lines=config["split_lines"],

threshold=config["threshold"]

priority: 70

shell:

"mkdir $PWD/de_novo_prediction && cd $PWD/de_novo_prediction && python ../src/de_novo_prediction.py -g {input.genome} -l {params.split_lines} -t {params.threshold} -o {params.prediction_out}"

rule batch_submit_job:

params:

run_type=config["run_type"],

batch_submit_jobs_number=config["batch_submit_jobs_number"],

prediction_out=config["prediction_out"]

priority: 60

shell:

"cd src/ && bash batch_submit.sh {params.run_type} {params.prediction_out} {params.batch_submit_jobs_number}"

rule cat_predict:

priority: 50

shell:

"cd src/ && bash cat_predict.sh"

rule get_positive_sample:

params:

prediction_out=config["prediction_out"],

speices_name=config["speices_name"]

priority: 40

shell:

"python src/get_positive_sample.py -i {params.prediction_out} -n {params.speices_name}"

rule bowtie2_index:

input:

genome=expand("{sample}", sample=config["genome"])

params:

index_name=config["index_name"]

priority: 30

threads: 16

shell:

"mkdir $PWD/peak && cd $PWD/peak && bowtie2-build {input.genome} {params.index_name}"

rule bowtie2:

params:

index_name=config["index_name"],

sam_prefix=config["sam_prefix"]

priority: 20

threads: 16

shell:

"cd $PWD/peak && bowtie2 -x {params.index_name} -f ../de_novo_prediction/whole_pre.fa -S {params.sam_prefix}.sam"

rule macs2:

params:

sam_prefix=config["sam_prefix"],

peak_prefix=config["peak_prefix"]

priority: 10

threads: 16

shell:

"cd $PWD/peak && source activate macs2 && macs2 callpeak -t {params.sam_prefix}.sam -f SAM --shift -125 --extsize 250 --nomodel -B --SPMR -g hs -n {params.peak_prefix}"
