# Supplementary figures and images for: CharPlant: A De Novo Open Chromatin Region Prediction Tool for Plant Genomes
Source: Genomics Proteomics Bioinformatics. 2021 Mar 2;19(5):860–71. doi: 10.1016/j.gpb.2020.06.021 (PMC9170768; doi:10.1016/j.gpb.2020.06.021)

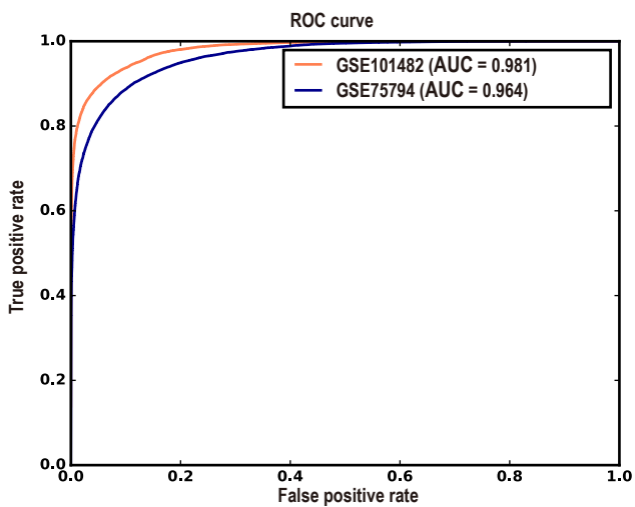

Supplement: Supplementary Figure S1 — ROCs of Oryza sativa ATAC-seq data ROC, receiver operating characteristic; AUC, area under the ROC curve. [file mmc2.pdf]

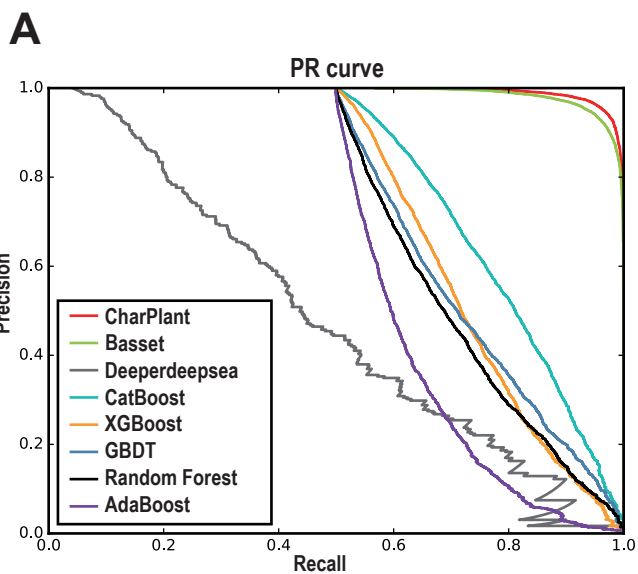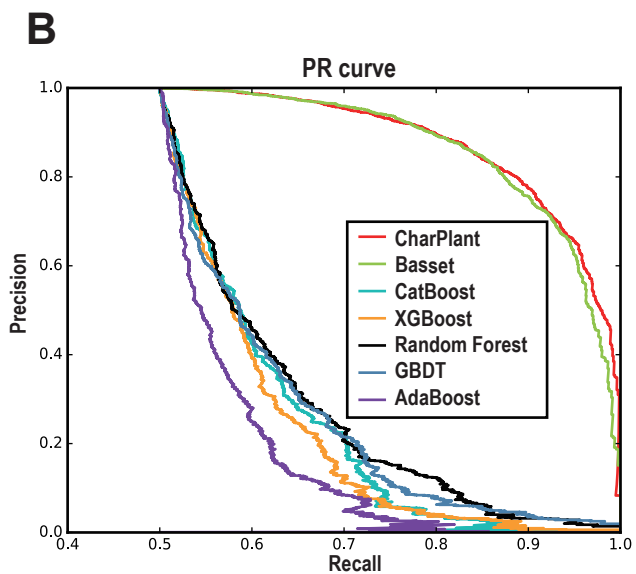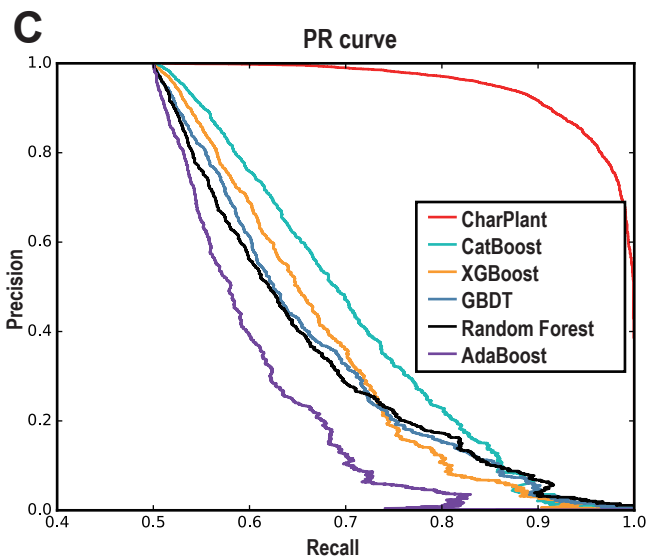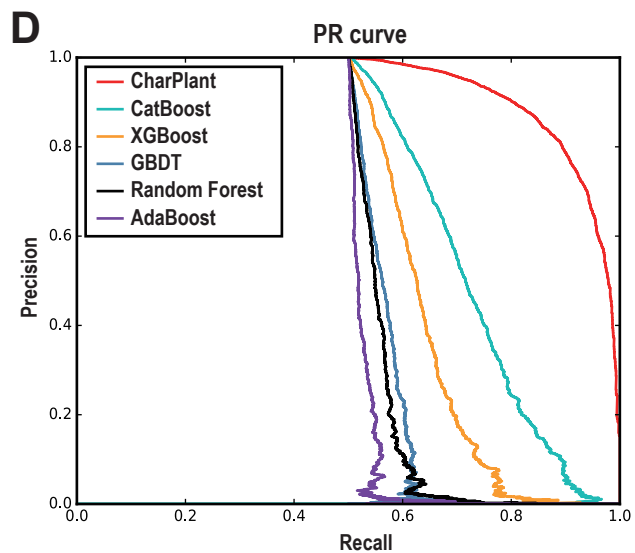

Supplement: Supplementary Figure S2 — Precision recall curves of comparative methods in four plant species A.Oryza sativa. B.Arabidopsis thaliana. C.Medicago truncatula. D.Solanum lycopersicum. PR, precision recall. [file mmc3.pdf]

**A**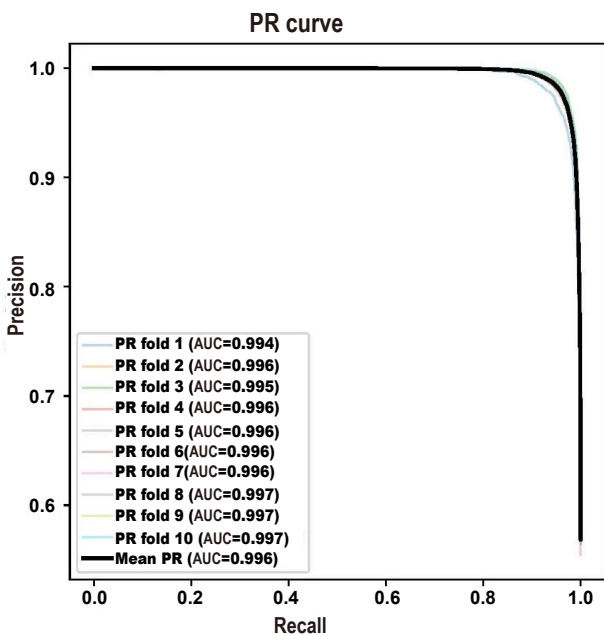**B**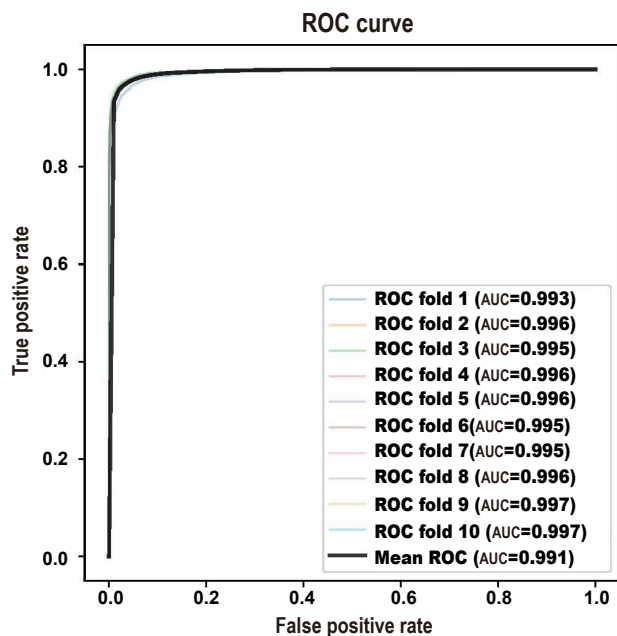

Supplement: Supplementary Figure S3 — Receiver operating curves and precision recall curves of 10-fold cross validation A. Receiver operating curves. B. Precision recall curves. ROCs, receiver operating curves; PR, precision recall. [file mmc4.pdf]

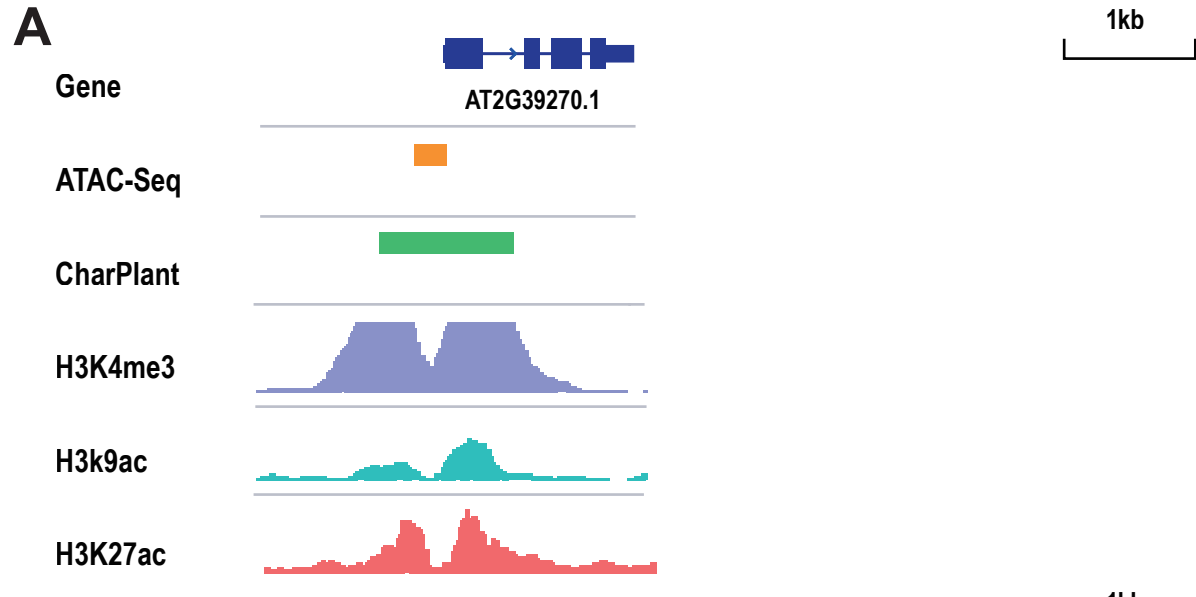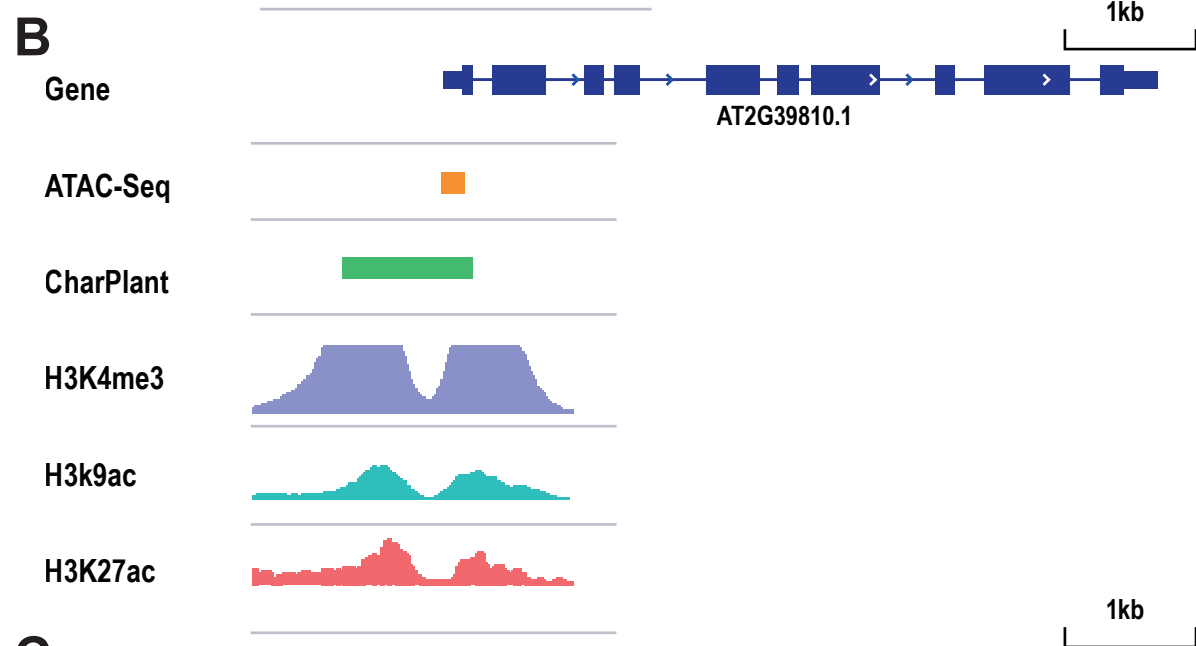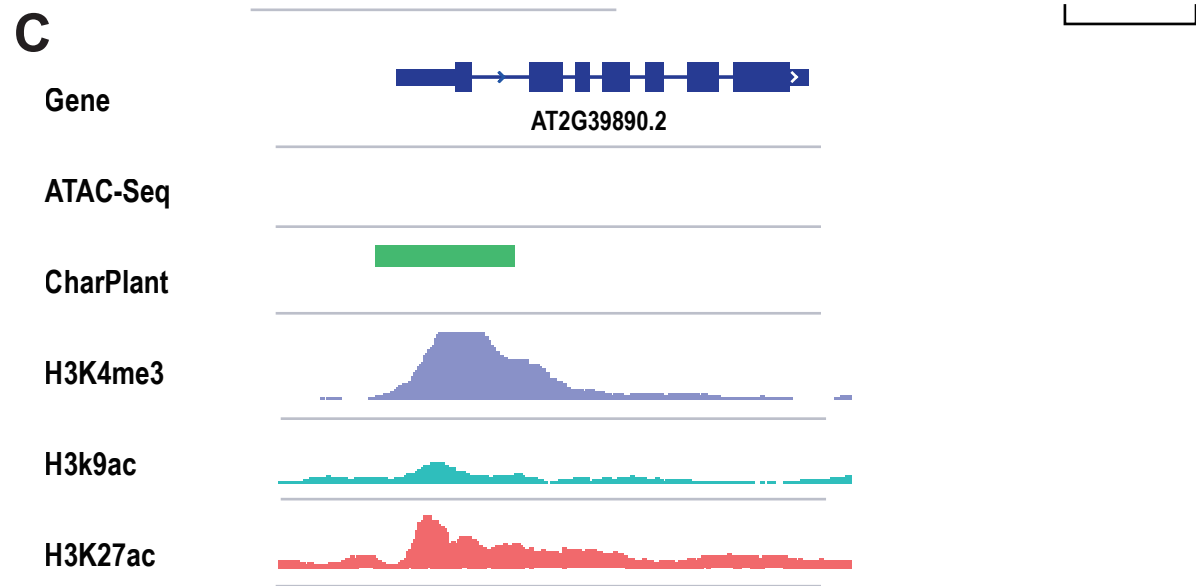

Supplement: Supplementary Figure S4 — Comparison of predicted OCRs, experimental OCRs, and histone modifications H3K4me3, H3K9ac, and H3K27ac in Arabidopsis thaliana A. and B. Two examples showing overlap between predicted OCRs and experimental OCRs. C. An example where ATAC-seq data showed no peak at the predicted OCR, but H3K4me3, H3K9ac, and H3K27ac modifications showed significant peaks. OCR, open chromatin region. [file mmc5.pdf]

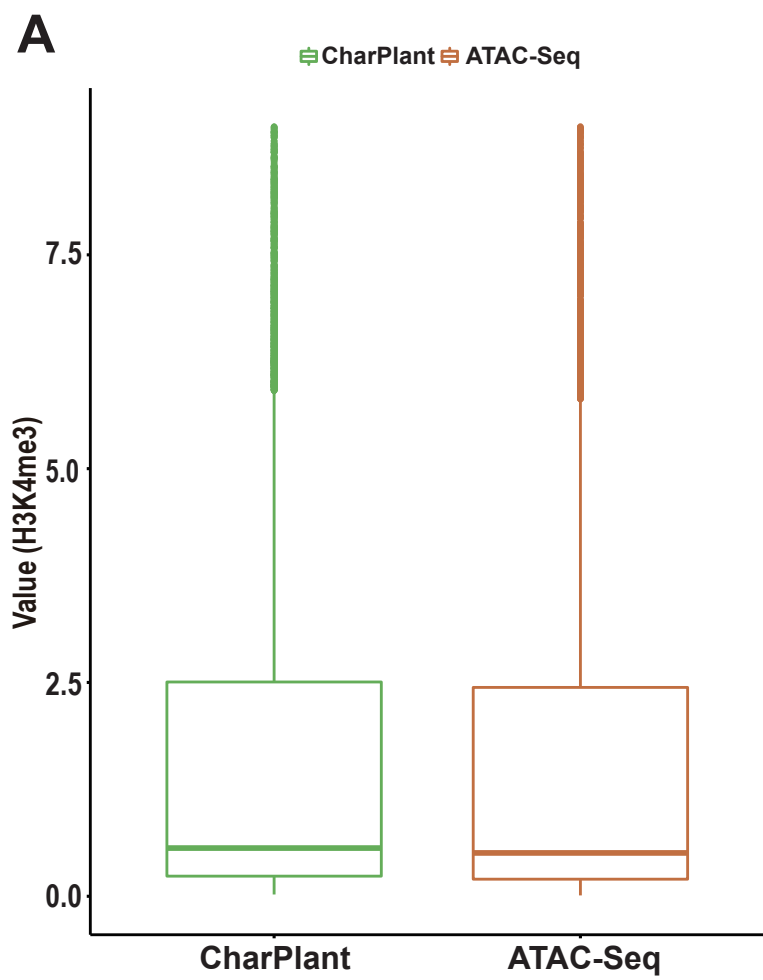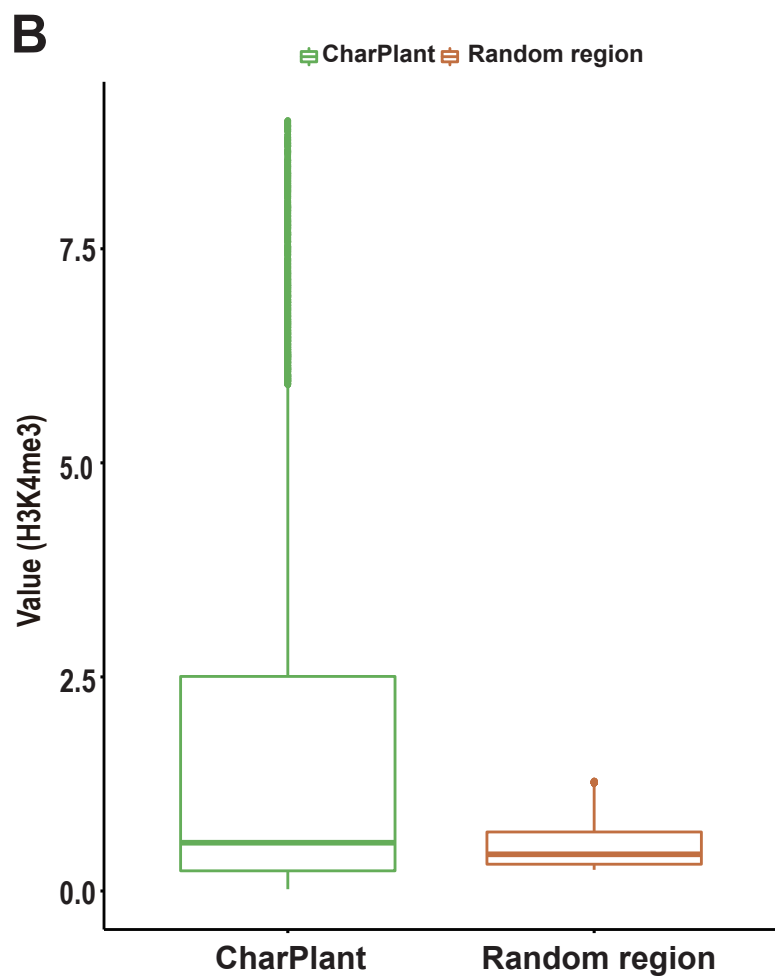

Supplement: Supplementary Figure S5 — Difference in the epigenetic modification H3K4me3 in Arabidopsis thaliana A. Between the open chromatin regions predicted by CharPlant and ATAC-seq peaks. B. Between the open chromatin regions predicted by CharPlant and randomly selected inactive regions. [file mmc6.pdf]
